# Data-Driven Estimation of a Driving Safety Tolerance Zone Using Imbalanced Machine Learning

**DOI:** 10.3390/s22145309

**Published:** 2022-07-15

**Authors:** Thodoris Garefalakis, Christos Katrakazas, George Yannis

**Affiliations:** Department of Transportation Planning and Engineering, National Technical University of Athens, 5 Iroon Polytechniou Str., 157 73 Athens, Greece; theogar@windowslive.com (T.G.); geyannis@central.ntua.gr (G.Y.)

**Keywords:** driving behavior analysis, driving behavior classification, imbalanced machine learning

## Abstract

Predicting driving behavior and crash risk in real-time is a problem that has been heavily researched in the past years. Although in-vehicle interventions and gamification features in post-trip dashboards have emerged, the connection between real-time driving behavior prediction and the triggering of such interventions is yet to be realized. This is the focus of the European Horizon2020 project “i-DREAMS”, which aims at defining, developing, testing and validating a ‘Safety Tolerance Zone’ (STZ) in order to prevent drivers from risky driving behaviors using interventions both in real-time and post-trip. However, the data-driven conceptualization of STZ levels is a challenging task, and data class imbalance might hinder this process. Following the project principles and taking the aforementioned challenges into consideration, this paper proposes a framework to identify the level of risky driving behavior as well as the duration of the time spent in each risk level by private car drivers. This aim is accomplished by four classification algorithms, namely Support Vector Machines (SVMs), Random Forest (RFs), AdaBoost, and Multilayer Perceptron (MLP) Neural Networks and imbalanced learning using the Adaptive Synthetic technique (ADASYN) in order to deal with the unbalanced distribution of the dataset in the STZ levels. Moreover, as an alternative approach of risk prediction, three regression algorithms, namely Ridge, Lasso, and Elastic Net are used to predict time duration. The results showed that RF and MLP outperformed the rest of the classifiers with 84% and 82% overall accuracy, respectively, and that the maximum speed of the vehicle during a 30 s interval, is the most crucial predictor for identifying the driving time at each safety level.

## 1. Introduction

Road safety is a matter of major concern and significantly affects people worldwide. According to the World Health Organization (WHO), road accidents are the 8th leading cause of death for people of all ages and the 1st leading cause for people aged between 5 and 29 years old [1]. Worldwide, approximately 1.3 million human lives are lost each year, with significant consequences for society. As a result, the European Union and World Health Organization have set a goal of reducing fatal road accidents by 50% for the decade 2021–2030, with a special emphasis on the contribution of new technologies in the field of road safety.

Generally, road safety is affected by many different risk factors such as the driver’s state and environmental and traffic conditions [2]. However, human error still has a major contribution to traffic collisions [3]. The continuous development in the field of automatic vehicles aims to improve road safety, excluding the human element from the task of driving [4]. In addition, the use of intelligent driving behavior monitoring systems for real-time interventions proved to be particularly effective in improving road safety [5].

In recent years the research community has had a crucial role in the evolution of Intelligent Transportation Systems (ITS) and specifically of Connected and Automated Vehicles (CAVs). Several published studies focus on understanding the effect of different characteristics on dangerous driving to develop the right models for recognizing risky driving behavior while setting the framework for in-vehicle interventions. Although a variety of in-vehicle and post-trip interventions have been proposed [6,7], there is a lack of intervention personalization and a direct link between real-time driving behavior and the triggering of interventions. In recent years, driving behavior analysis by utilizing machine learning techniques has been of high interest to the research community [8]. 

The objective of the European Commission Horizon2020 project i-DREAMS (https://idreamsproject.eu/) is to define, develop, test, and validate a ‘Safety Tolerance Zone’ (STZ) in order to ensure safe driving behavior [9]. Through real-time monitoring of risk factors related to task complexity (e.g., traffic characteristics and weather) and coping capacity (e.g., driver’s mental state, driving behavior and vehicle current state), i-DREAMS aims to identify the level of STZ and to develop interventions in order to keep the driver within acceptable boundaries of safe operation. The STZ is divided into three levels: ‘Normal’, ‘Dangerous’ and ‘Avoidable Accident’. ‘Normal’ refers to the scenario that a crash is unlikely to occur, while ‘Dangerous’ concerns the increased possibility of crash occurrence, however, the accident is not inevitable. Lastly the ‘Avoidable Accident’ level of the STZ refers to a high possibility of crash occurrence but there is still time for the drivers to intervene in order to avoid a crash. The difference between the ‘Dangerous’ and Avoidable Accident’ level is that the need for action is more urgent in the ‘Avoidable Accident’ level. 

However, linking driving characteristics to latent concept of risk or defining risk through different levels of driving behavior is a demanding task for road safety experts. Furthermore, the imbalance of road safety datasets is a well-documented problem and poses another obstacle to the correct identification of safety levels from driving behavior data. As a result, in the context of the present research, these challenges are attempted to be efficiently tackled.

Based on the aforementioned gaps in recent literature on real-time interventions and the prediction of driving behavior, this study aims to apply machine learning techniques to identify the level of the STZ concerning dangerous driving behavior and predict the duration of the time interval, which each driver spends at each level of risk, based on significant driving behavior indicators. In summary, this research proposes a framework for (a) defining the STZ levels, and (b) developing and evaluating machine learning algorithms to classify driving behavior and predict the duration that each driver spends at each risk level. This framework also exploits the most important features to identify driving behavior and takes care of dataset imbalance, which is a common problem in road safety analyses [10]. The paper contributes to the current knowledge in a two-fold matter; initially by identifying the level of safety of drivers in real-time, which is a real-time classification problem, and consequently by predicting the duration of each safety level in real-time. In that way, practitioners and OEMs based on driving behaviour characteristics, weather and driver’s state could trigger the necessary real-time interventions according to the prevalent safety level and its corresponding duration and bring drivers back to safe conditions. Furthermore, the prediction of the duration at each STZ level is a new approach for real-time driving behaviour assessment and has not been developed in previous research. Finally, in this research, an extensive comparative analysis of the techniques used to deal with certain challenges of the driving behaviour analysis studies was performed. 

It should be mentioned here, that although the authors and the project partners within i-DREAMS have already published papers on the project, and the use of imbalanced learning, the majority of the papers are either literature reviews or concerned with the single task of classifying driving conditions. The present paper is one of the first attempts to exploit a data-driven approach to define the STZ and predict both the level and the duration of each corresponding level.

The paper is structured as follows: after the introduction, an extensive literature review is conducted on driving behavior analysis using machine learning techniques. This is followed by the description of the research methodology, which includes the theoretical background of the models. Then, the collection and processing of the dataset are described. Finally, the results of the analysis are presented, in order to draw conclusions, related to road safety.

## 2. Literature Review

In recent years, the two main approaches that are widely used to analyze dangerous driving behavior are simulator studies and naturalistic driving studies (NDS) [11]. According to [12], the severity of dangerous driving is related to certain traffic, driving, vehicle, and environmental factors. Furthermore, recent studies focus on identifying driving behavior and classifying it as dangerous or safe since the real-time prediction of the safety level can trigger interventions and consequently improve road safety [13]. In a more anthropocentric approach, studies have developed models to evaluate dangerous driving behavior based on the driver’s state [14] and based on certain characteristics of the driver, such as demographics [15]. Other studies have developed models of recognizing dangerous driving based on driving behavior parameters such as speed, time to collision, and time to headway [13,16,17].

Risky driving behavior prediction models based on machine learning algorithms have become extremely popular, due to their high scoring accuracy. In relevant studies, the most utilized models with high performances were Random Forest (RFs; [15]), Multilayer Perceptron (MLP; [16]), Support Vector Machines (SVMs; [13]) and eXtreme Gradient boosting (XGBoost; [17]). For instance, [16] proposed a methodology to predict and evaluate the risk of the driver in real-time, based on four safety levels of driving behavior. In this study, the proposed methodology includes feature extraction, clustering techniques, feature importance, and the development and evaluation of four machine learning algorithms (i.e., RF, XGBoost, SVM, and MLP) where accuracy is higher than 85%. [13] applied a methodology to classify and evaluate different risk levels of driving behavior by analyzing a driving simulator dataset, developing clustering techniques in order to distinguish the different levels, and applying two classification algorithms (i.e., SVM and Decision Tree) with the highest accuracy to be 95%. Moreover, [17] proposed a framework for risk prediction which includes applying feature selection techniques, risk levels labeling, developing methods to deal with imbalanced datasets, and evaluating a classification model (i.e., XGBoost) with an overall accuracy of 89%. 

Labeling and distinguishing safety levels is a topic that has become of interest for many researchers in the past as it is a demanding process and an important one for the development of Advanced Driver Assistance Systems (ADAS). In previous studies, determination and evaluation of different risk levels of driving behavior have been accomplished based on several safety indicators, such as time to collision [18]. However, it is difficult to set the right thresholds for different risk indicators making the process of defining safety levels problematic [17]. As an alternative, some researchers have proposed a framework for determining the different risk levels by utilizing several clustering techniques, such as k-means and hierarchical clustering [13,16,19]. 

Furthermore, since the analysis of driving behavior is based on a real-world dataset, there is a data imbalance problem in all previous studies in terms of their distribution in each class (i.e., safe and dangerous conditions). Specifically, in the relevant studies, dangerous behavior and the possibility of an accident are rarer in relation to safe driving behavior and non-accident, respectively. The class with the most data is called the majority class while the one with the least data is called the minority class. In real-time collision analysis problems, the ratio of the crash and non-crash ranges from 1:5 [20] to 1:20 [21]. The most common sampling techniques in the literature are the Synthetic Minority Oversampling Technique (SMOTE) [16,22,23,24] and Adaptive Synthetic (ADASYN) [24]. In addition, based on the literature review in the field of road safety as well as different scientific areas, additional sampling techniques tend to be efficient methods such as the combination of SMOTE and Edited Nearest Neighbors (SMOTE-ENN) [10], Random Oversampling, SVM-SMOTE and SMOTE-Tomek [25]. 

In general, most previous studies on driving behavior analysis have focused on developing a specific framework for identifying risky driving behavior. An alternative approach is to predict the duration of driving at the different safety levels. In the framework of the research project i-DREAMS, [9] propose the prediction of continuous indicators of risk such as the time spent at each safety level in order to tune the frequency of warnings triggered to the driver in real-time. Although to our knowledge, a similar development of the above approach has not been found in research, a similar methodology is applied to short-term traffic prediction problems [26,27,28]. 

## 3. Methodology

### 3.1. Definition of STZ Level

As the primary aim of this research is to identify the risk level of driving behavior, i.e., the level of the STZ, it is important to identify the best way to define these different safety levels. After a brief literature review, the number of different driving safety levels was determined to be three, with labels ‘Normal’, ‘Dangerous’, and ‘Avoidable Accident’. The above three levels are defined using two groups of methods: (i) clustering methods (e.g., K-means, Hierarchical, etc.) and (ii) threshold-based methods (e.g., a threshold of Speed, Time to Collision, Time Headway, etc). 

The main limitation is that the distribution of the dataset must comply with the available literature, in which dangerous driving behavior occurs less frequently. Specifically, the ‘Normal’ level must be the major class with the highest percentage of samples, while the ‘Dangerous’ and ‘Avoidable Accident’ levels must be the minority class with the lowest percentage of samples.

### 3.2. Feature Selection

An important step in the classification process is to perform a feature selection. Feature selection refers to the process of reducing the number of input variables to reduce computational complexity and prediction errors [22]. Based on the literature review, two approaches are proposed, (i) correlation-based feature selection [29], and (ii) permutation importance-based feature selection [30]. 

The first approach concerns the determination of the correlation between the independent variables based on the Pearson correlation coefficient r. The values of the coefficient range between −1 and 1, where r = 0 refers to zero correlation, r = 1 to full positive correlation, and r = −1 to full negative correlation. The optimal subset consists of characteristics quite correlated with the predicted class but having minimal correlation between them [29]. 

The second approach attempts to measure the importance of input variables in the classification process by permuting the feature and calculating the increase in the model’s prediction error. 

### 3.3. Imbalanced Learning

As indicated in the literature review, dangerous driving behavior is a rarer phenomenon than normal driving behavior. In addition, the fact that classification algorithms work by considering the equal distribution of samples in different classes; the research has some limitations. In this study, the methods of improving the performance of the models will be discussed and analyzed in order to deal with the bias of algorithms towards the majority class.

After the brief literature review, many resampling methods were examined, such as SMOTE, SMOTE-ENN, etc. However, the Adaptive Synthetic (ADASYN) technique, an improved version of the Synthetic Minority Oversampling Technique (SMOTE), is considered the most suitable for handling imbalanced datasets and avoiding overfitting [24,31,32]. The main idea behind the ADASYN algorithm is the use of difficulty in learning for different minority examples as a criterion to determine the appropriate number of synthetic samples that need to be generated for each minority data example [33]. In addition, after examining individual resampling techniques, ADASYN contributed to the highest performance in the classification process compared to the rest (i.e., SMOTE, SMOTE-ENN, SVM-SMOTE, SMOTE-Tomek and Random Oversampling).

### 3.4. Multiclass Classification

As the objective of this study is to identify the driving behavior risk level between three classes (i.e., Normal, Dangerous, Avoidable Accident), the problem is a multi-class classification. The proposed method is based on certain risk-driving indicators as predictor variables and four different machine learning classification algorithms: (i) Support Vector Machines, (ii) Random Forest, (iii) AdaBoost, and (iv) Multilayer Perceptron. 

The four classification algorithms were proposed due to their high performance and common use on literature for dangerous driving behavior identification, for real-time crash prediction and for other real-world problems.

To train and evaluate the performance of classification algorithms, the dataset is divided into a training dataset and a testing dataset. The form of a training dataset is X_training_ = {(x_n_, y_n_), *n* = 1, N}, where x_n_ is a predictor variable and y_n_ = {0,1,2} is the target variable. By training the model, it is given the ability to classify new data correctly. The performance of the classification model is easily illustrated through a confusion matrix, where one axis represents the actual class while the other the predicted class. The results demonstrated in this paper, were obtained after utilizing 10-fold cross validation. 

The classification algorithms are evaluated using the accuracy, precision, recall, f1-score, and false alarm rate defined by Equation (1) to Equation (5).

(1) $$\mathrm{Accuracy}=\frac{TP+TN}{TP+FP+FN+TN}$$

(2) $$\mathrm{Precision}=\frac{TP}{TP+FP}$$

(3) $$\mathrm{Recall}=\frac{TP}{TP+FN}$$

(4) $$f1-\mathrm{score}=\frac{2\times \left(Precision\right)\times \left(Recall\right)}{\left(Precision\right)+\left(Recall\right)}$$

(5) $$\mathrm{False}\;\mathrm{Alarm}\;\mathrm{Rate}=\frac{FP}{FP+TN}$$

where: True Positive (*TP*) represents the instances which belong to class i and were correctly classified in it; True Negative (*TN*) represents the instances which do not belong to class i and were not classified in it; False Positive (*FP*) represents the instances which do not belong to class i but were incorrectly classified in it; False Negative (*FN*) represents the instances which belong to class i but were not classified in it.

The accuracy metric calculates the percentage of instances which were correctly classified. In problems with an imbalanced dataset, the ‘Accuracy Paradox’ is observed where the calculated accuracy is affected by the major class without reflecting the actual situation [34]. The precision metric shows the percentage of data that actually belongs to class i of all the data that the model classified in class i., while recall describes the percentage of data that actually belongs to class i and the algorithm was able to classify them correctly in class i. In this study, the effects of incorrectly classifying a risk class as less risky or safe would have significant consequences on road safety, making recall a powerful evaluation metric. Lastly, f1-score represents the harmonic measure between precision and recall while the false alarm rate resembles the probability of false detection.

### 3.5. Classification Algorithms

The four classification algorithms as described in Section 3.4 are (i) Support Vector Machines, (ii) Random Forest, (iii) AdaBoost, and (iv) Multilayer Perceptron.

#### 3.5.1. Support Vector Machines (SVM)

SVM are supervised learning models that can be useful for classification and regression problems [35]. The key idea is that SVM tries to find the maximum margin hyperplane while minimizing the distance between misclassified instances and decision boundaries [36]. Also using the kernel method, SVM can manage nonlinearly separable data. Based on literature, SVM algorithm has been used extensively in road safety studies and has been shown to achieve high performance [13]. Furthermore, SVMs have the advantage to handle high-dimensionality datasets [37]. 

Utilizing the hyperparameter tuning technique Grid Search, the optimal values of the SVM’s hyperparameters were obtained. The most important SVM’s hyperparameters emerged through GridSearchCV from scikit-learn python’s library, were: (a) kernel type = ‘rbf’; (b) regularization parameter C = 50 and (c) kernel coefficient gamma = ‘scale’.

#### 3.5.2. Random Forest (RF)

RF classifier is an ensemble method, which trains multiple decision trees in parallel utilizing the bootstrapping and aggregation methods, commonly known as the bagging technique [38]. The bootstrapping technique is described as the parallel training of multiple decision trees using different subsets of datasets. The final decision results from the aggregation of the decisions of the individual decision trees. RF classifier tends to perform efficiently on classification tasks and more specifically on identifying risky driving behavior. Furthermore, RF benefits from the fact that can overcome overfitting problem of decision trees [16] and thus RF algorithm is considered a good choice for identification of risky driving behavior.

Grid Search was also used for the RF model, and the optimal hyperparameters that it obtained were: (a) the number of estimators/trees of the forest = 200 and (b) the function to measure the quality of a split (criterion) = ‘entropy’.

#### 3.5.3. AdaBoost

AdaBoost model is an ensemble method, which trains several decision trees in series. A set of weak classifiers are connected in series where each weak classifier tries to improve the classification of the samples that were incorrectly classified from the previous one; the method is known as boosting [38]. The weight of misclassified instances by the previous tree is boosted for the subsequent tree to classify them correctly. Based on the literature, AdaBoost is suitable for most types of data, and more specifically has high performance for imbalanced datasets avoiding overfitting issues. Furthermore, the training of multiple weak classifiers in order to form a synthetic classifier with high efficiency is much easier compared to the training of one strong classifier [39]. Therefore, since the present study concerns imbalanced dataset, it makes AdaBoost a good alternative.

Through GridSearchCV the optimal of maximum number of estimators was set to be 500.

#### 3.5.4. Multilayer Perceptron (MLP)

MLPs are neural network models and more specifically are a supplement of feedforward neural networks. Multilayer perceptron consists of three categories of layers: (i) the input layer which receives the input data that need to be processed, (ii) the hidden layers that are the computational power of the model and (iii) the output layer which perform the prediction of the classification process. MLP classifier is commonly used for pattern classification, recognition, prediction and approximation [40] and as stated previously has proven to be effective algorithm in driving behavior analysis studies [16].

The optimal hyperparameters that emerged from the Grid Search optimization for the MLP model were: (a) number of hidden layers = (500, 500, 500); (b) activation function = ‘relu’; and (c) alpha parameter of regularization term = 0.0001.

### 3.6. Multiple Linear Regression 

After defining the driver’s behavior risk level for each time frame of 30 s, the duration that each driver spends in each risk level was calculated by summing these time frames. In multiple linear regression, the purpose is to estimate the statistical significance and the relationship between a dependent variable (y) and multiple independent variables (x_i_) [41]. The effect of each independent variable on the dependent is expressed through coefficients of regression. In this study, an attempt is made to develop regression models to predict the duration that a driver spends at each safety level using certain driving behavior factors as the dependent variables. 

In order to evaluate the regression models, the coefficient of determination R^2^ (Equation (6)) is used, which calculates the percentage of the variance of the dependent variable (*y*) interpreted by the independent variables (x*_i_*). The coefficient of determination (R^2^) measures the ability of features to interpret a phenomenon and its values range from 0 to 1.

(6) $$R^{2}=\frac{\sum_{i=1}^{n}{\left(\hat{y}-\bar{y}\right)}^{2}}{\sum_{i=1}^{n}{\left(yi-\bar{y}\right)}^{2}}$$

Note: *n* is the number of samples; *y_i_* is the actual values of dependent variable *y*; $\bar{y}$ is the mean value of dependent variable y; ${\hat{y}}_{i}$ is the predicted values of dependent variable *y*.

To evaluate the effect of the independent variables, the logical explanation of the coefficients as well as the statistical significance of the variables were examined. When the null hypothesis is rejected at a significance level (a), the sample is characterized as statistically significant and suggests that the influence on the occurrence of the phenomenon is not due to chance. The statistical significance is evaluated by using *p*-value and t-value. For a *p*-value lower than the significance level (a) and for a t-value greater than the t-student distribution, the null hypothesis is rejected.

It is also important to note, that the selection of independent variables is made based on their correlation as well as their statistical significance in the development of the models.

### 3.7. Regression Algorithms

This study is based on three regression algorithms: (i) Ridge Regression, (ii) Lasso Regression, and (iii) Elastic Net Regression. These models benefit from their ability to deal with multicollinearity and their ability to perform a type of feature selection. The key idea behind these models is the regularization of least-squares by utilizing a regularization parameter λ [42]. The choice of the specific algorithms over machine learning regressors, such as Support Vector Regressor, was based on the need to investigate the influence of independent variables in the prediction process through coefficients.

#### 3.7.1. Ridge Regression

Ridge Regression is a regularization model which can deal high multicollinearity of independent variables. As stated previously, a regularization parameter *λ* is introduced to minimize the weight of regression coefficients (b) towards zero, reducing the variability of estimates. Through *λ* parameter Ridge Regression model can reduce the impact of non-important features in the prediction process. The regularization technique that Ridge Regression utilizes is called L_2_ regularization. The estimated coefficients (b) of Ridge Regression minimize the function Equation (7) [43]:

(7) $$\overset{n}{\underset{i=1}{\sum }}{\left(y_{i}-b_{0}-\overset{P}{\underset{j=1}{\sum }}b_{i}x_{ij}\right)}^{2}+\lambda \overset{P}{\underset{j=1}{\sum }}b_{\dot{J}}^{2}$$

#### 3.7.2. Lasso Regression

Lasso Regression (Least Absolute Shrinkage and Selection Operator) has many similarities with Ridge Regression since it also regularizes the cost function using a regularization parameter *λ*. However, Lasso Regression has the ability to select the most important independent variables ignoring those with minimal effect on the dependent variable. Using the L_1_ regularization technique, the coefficients of the least important variables tend to zero performing a selection of the most important features and dealing with model’s overfitting [44]. The estimated coefficients (b) of the Lasso regression minimize the function Equation (8) [43]:

(8) $$\overset{n}{\underset{i=1}{\sum }}{\left(y_{i}-b_{0}-\overset{P}{\underset{j=1}{\sum }}b_{i}x_{ij}\right)}^{2}+\lambda \overset{P}{\underset{j=1}{\sum }}\left|bj\right|$$

#### 3.7.3. Elastic Net Regression

Elastic Net Regression [45] is the combination of Ridge and Lasso regression. It is a highly efficient algorithm as it combines the abilities and the benefits of both Ridge and Lasso by utilizing two regularization parameters. The estimated coefficients (b) of the Lasso regression minimize the function Equation (9):

(9) $$\sum_{i=1}^{n}{\left(y_{i}-b_{0}-\sum_{j=1}^{P}b_{i}x_{ij}\right)}^{2}+\lambda_{1}\sum_{j=1}^{P}\left|bj\right|+\lambda_{2}\sum_{j=1}^{P}b_{\dot{J}}^{2}$$

## 4. Data Collection and Processing

### 4.1. Data Collection

**Figure 1:**
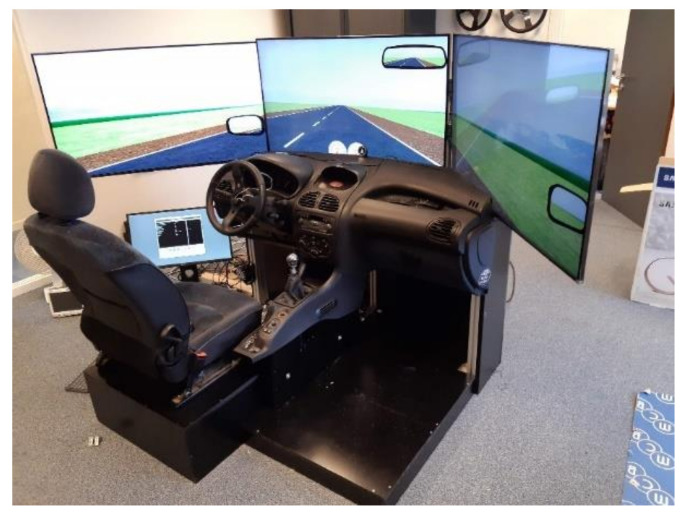
DSS Car Simulator.

The study is based on the hypothesis that driving behavior is affected by different risk factors. As part of the i-DREAMS research project, 36 drivers participated in a driving simulator experiment to collect important data on various risk factors. The experiment was conducted from December 2020 to January 2021, using the DSS driving simulator of Figure 1 which was designed and built for the i-DREAMS project.

Three different driving scenarios were implemented. These scenarios aim to assess the impact of interventions on road safety in real-time. The simulator trials in i-DREAMS were designed based on several principles derived from previous literature [46,47] including definition of outcomes, predictors and hypotheses, selection of sample size and statistical power, selection of design type, distribution of risk scenarios among participants, selection of drive durations to avoid simulator sickness, avoidance of order and learning effects, and consideration of confounding effects. Of course there are limitless alternatives for designing simulator trials, but the details described in Table 1 were deemed the best from project partners with regards to the outcomes of the project.

Each participant performed three separate drives.

- Drive 1: No interventions
- Drive 2: Interventions
- Drive 3: Interventions with modifying condition

The variables collected from the driving simulator experiment are described in Table 2. The collected variables are important risk factors for driving behavior related to traffic conditions and the driver’s state.

### 4.2. Data Processing

To simplify the process, the data were aggregated in 30 s intervals. More specifically, for every 30 s interval, descriptive statistics of each variable such as mean value, standard deviation, minimum value, maximum value, and median were calculated.

#### 4.2.1. Definition of Driving Behavior Risk Level

An initial step before developing classification and regression models is to determine the different safety levels of driving behavior. For the identification of driver’s behavior level, it was important to determine the level of road safety. As it is stated in Section 3.1 and based on the literature review the three clusters are defined examining the two following groups of methodologies: (i) clustering methods (e.g., K-means, Hierarchical, etc.) and (ii) threshold-based methods (e.g., a threshold of Speed, Time to Collision, Time Headway, etc.). Regarding the first group, by examining some clustering techniques, the results were not satisfactory. The distribution of the samples in the different classes (i.e., ‘Normal’, ‘Dangerous’ and ‘Avoidable Accident’ level) was not in line with the literature, considering the ‘Avoidable Accident’ safety level as the major class while the ‘Normal’ safety level as the minority class. As stated previously, in relevant studies, risky driving behavior is a rarer phenomenon compared to safe driving behavior, and therefore should represent the class with the minority of samples.

So, in order the study analysis to be consistent with the literature, meaning the samples of dangerous driving to be a minority class, threshold-based methods (e.g., a threshold of Speed, Time to Collision, Time Headway, etc.) were examined. Table 3 shows the distribution of samples in different classes based on different techniques of safety level determination.

Therefore, the threshold method for the variable Headway_min provided the most desired results. According to previous research, a time of headway of 1.1 to 1.7 s is considered a tolerable margin [48]. However, when the time headway is less than 2 s driving becomes more difficult and more dangerous [49]. In addition, several driver training programs state that 2 s is the minimum time distance from the vehicle in front in order to maintain a safe follow-up and avoid accidents [50]. Based on the above conclusions, for each level, the value range of the Headway_min variable is:

- ‘Normal’ Level: Headway_min > 2 s
- ‘Dangerous’ Level: Headway_min > 1.4 sec and Headway_min < 2 s
- ‘Avoidable Accident’ Level: Headway_min < 1.4 s

To avoid bias, the variables of Headway and TTC were excluded in the development of classification models.

#### 4.2.2. Feature Selection

As stated in Section 3.2, to minimize the computational cost and improve the predictive performance of the classification models, a feature selection was performed in which the number of input variables was reduced. The selection of input variables was made based on the correlation between variables and the influence of each variable in the classification process. 

**Figure 2:**
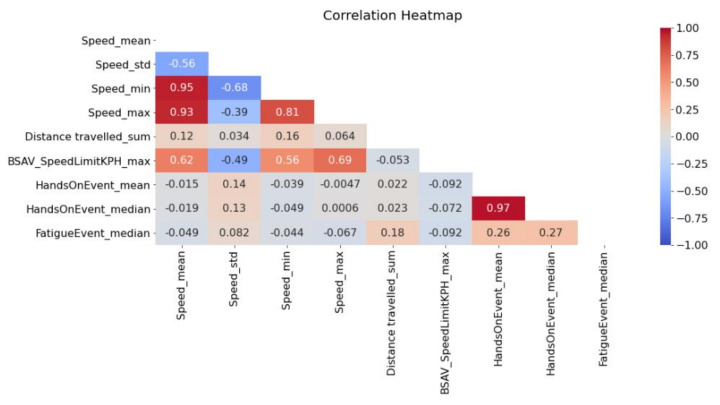
Correlation heatmap of the examined variables.

As it appears in Figure 2, a high correlation was observed between descriptive statistics of the same variable. Furthermore, Speed and Speed Limits (BSAV_SpeedLimit) were moderately correlated while HandsOnEvent, FatigueEvent, Distance travelled and all other variables were slightly correlated.

**Figure 3:**
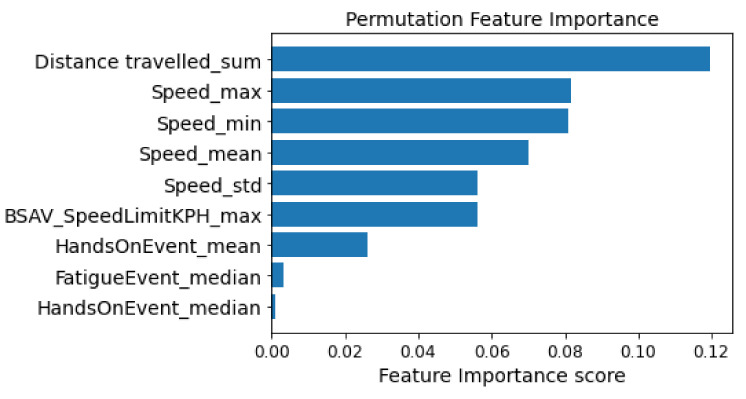
Permutation feature importance.

To identify the importance of the variables in the classification process, the permutation feature importance technique was used. As stated previously, the Permutation Feature Importance procedure calculates the prediction error after permuting the value of the feature. This technique breaks the relationship between the feature and the target; therefore the model’s prediction error after permutation of the feature’s value indicates whether or not the model depends on the feature [51]. An advantage of Permutation Feature Importance is the fact that there is no need for retraining the model which can save significant amounts of time. Also, a benefit of this technique is the fact that it takes into account all interactions with other features [52]. Based on Figure 3, the distance travelled, the speed and the speed limits have the greatest influence on the process of recognizing the safety level where the driver is. In contrast, the variables HandsOnEvent and FatigueEvent have the lowest impact on the classification process.

Based on the correlation and the feature importance that emerged, the input variables in the classification models are Distance travelled_sum, Speed_max, and BSAV_SpeedLimitKPH_max. Table 4 provides some descriptive statistics (i.e., mean value, standard deviation, maximum, minimum value, and maximum value) for input variables in the classification process.

## 5. Results

### 5.1. Evaluation of Identification Models of Risky Driving Behavior

As mentioned previously the four developed classification models to identify the driver’s safety level were, Support Vector Machines (SVM), Random Forest (RF), AdaBoost, and Multilayer Perceptron (MLP). Due to the imbalanced dataset and based on the literature for metrics in imbalanced learning problems [34], the accuracy would provide misleading results. Accuracy is influenced by the majority class and fails to reflect the real situation resulting in the phenomenon called “accuracy paradox”. For this reason, as shown in Table 5, additional evaluation metrics are considered such as Precision, Recall, f1-score, and False Alarm Rate.

**Figure 4:**
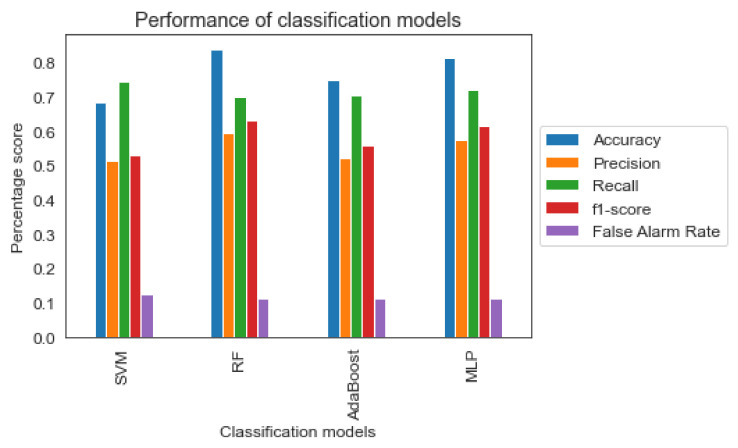
Classification metrics of the four machine learning models.

Observing Figure 4 the four algorithms score high accuracy and recall compared to precision and f1-score. However, as stated before for the current study, the recall metric is more important than precision as the incorrect identification of risky driving behavior into less risky would have serious implications for road safety. Especially for the “Avoidable Accident” safety level, the high recall combined with a lower precision rate implies a high ability to recognize the actual dangerous level but also implies a higher percentage of incorrect classification of the “Normal” and “Dangerous” levels as “Avoidable Accident”. In the context of the specific issue examined by this study, the above scenario is acceptable. In case of opposite results, there would be serious problems concerning road safety.

**Figure 5:**
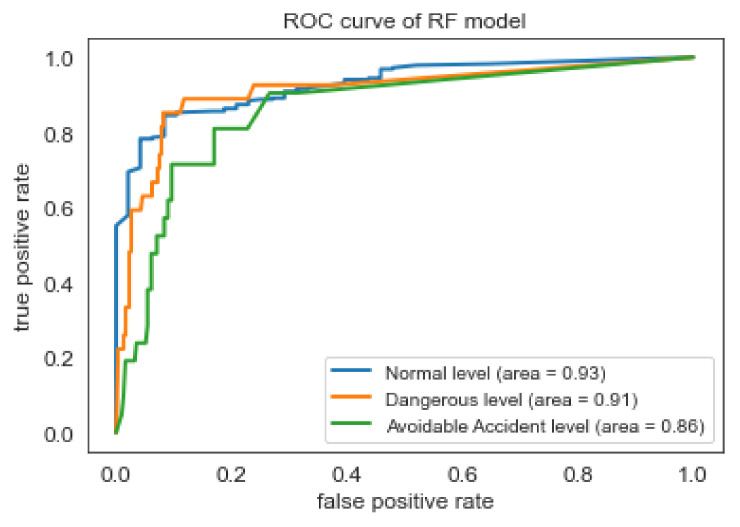
ROC curve of RF classifier.

**Figure 6:**
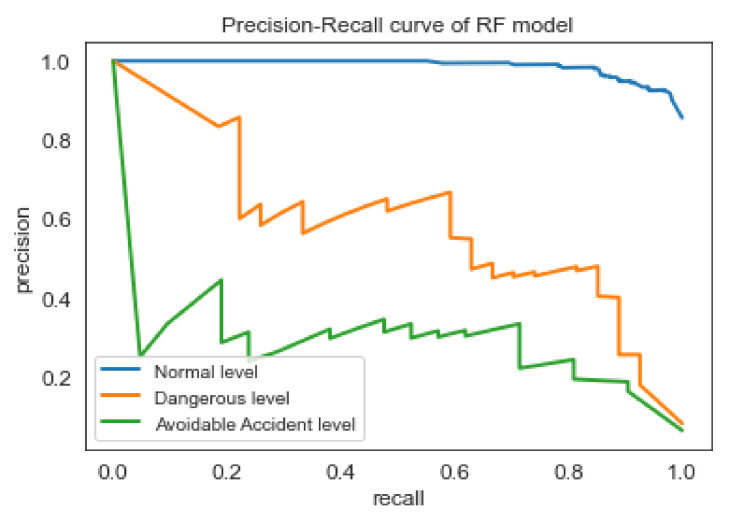
Precision-Recall curve of RF classifier.

Based on the Accuracy, Recall, and False Alarm Rate of the four models, the best results are offered by the RF and MLP classifiers. Nevertheless, the RF model performs slightly better than MLP, according to the f1-score of Table 5. As shown in the ROC curve of Random Forest classifier in Figure 5, the model seems to have high ability (approximately 90%), to distinguish between positive class and negative class for all three classes (i.e., safety levels). However, as found in the literature review [53], the interpretation of the ROC curves can be misleading especially in imbalanced classification problems. Precision-Recall curves, on the other hand, can provide a more realistic interpretation of the predictive power of the model. As shown in the Figure 6, for the different thresholds the Random Forest classifier seems to have better predictive ability for ‘Normal’ class comparing with the two other classes.

Based on similar driving behavior studies, the results of this research were realistically close with those found in the literature. Specifically, comparing the evaluation metrics of the RF classifier with those in the literature, it turns out that the performance of the model in this study had similar results. For example, [54] reached 71% of the actual conflict prediction, with 10% false alarm rate, whereas in this paper, the RF classifier reached 70% with 11% of false alarms. The only exception is the research of [15], where the percentage of correct classifications for the RF classifier was 90%, performing significantly better than the respective results of this research. The difference in the performance of the RF model between the present research and the [15] research may be due to the different nature of the characteristics considered as input variables, where the former takes into account characteristics of driving behavior while the latter analyzes characteristics such as gender, age and perception of the driver. Furthermore, as this study exploited data from a simulator, the identification of safety-critical levels might not be as clear as in real-life situations or naturalistic driving conditions. Additionally, the layers of the STZ were defined based on pre-defined threshold and not according to a data-driven method. As a result, the difficulty of classifiers on identifying correctly both safe and dangerous driving behavior might be hindered by that fact. A larger dataset and the utilization of more sophisticated clustering approaches (e.g., t-SNE) could overcome this limitation. Regarding the SVM classifier, the literature results outperformed those in this study, with the research of [13] achieving 95% accuracy. Furthermore, regarding the MLP classifier, this study had similar results concerning the accuracy metric compared to the research of [16]. However, the developed MLP classifier in the literature outperformed the one in this research, since the f1-score between the two studies had a significant difference of 20%. Lastly, although the application of AdaBoost was not found in the literature on the topic of driving behavior analysis, it had a satisfactory performance compared to the other classifiers found in the literature.

Although, the developed models might lack the utilization of more sophisticated models such as deep learning, they can be exploited by researchers and practitioners working in real-time crash risk assessment due to the fact that they were found to work well with the imbalance of the dataset and the use of highly disaggregated (i.e., 30 s) data. 

### 5.2. Evaluation of Prediction Models of Driving Duration in Each Safety Level

As stated in Section 3.7, based on the identification of the driver’s safety level for every 30 s interval, the total duration spent in each level is calculated by summing the time frames. Aiming to correlate the various variables with duration, their average value was calculated for each driver at each safety level.

Evaluating the performance of the models, the statistical significance, and the correlation between the variables, ‘Speed_max’ and ‘Distance travelled_sum’ were selected as independent variables. In contrast to the classification process, the variables of time to collision and time headway were also examined. The main aim was to develop regression models with statistically significant variables. Three regression algorithms were developed, Ridge Regression, Lasso Regression, and Elastic Net Regression and their results are demonstrated in Table 6, Table 7 and Table 8.

Based on the results of regression models, it is evident that the models record high values of R^2^, meaning the independent variables have a high ability to interpret the variance of the dependent variable. Taking into account the regression coefficients and the fact that the three models perform some kind of feature selection by minimizing the coefficient of the non-significant variables, it appears that the ‘Speed_max’ factor has the highest effect on the driving duration at each safety level. The negative coefficient of the ‘Speed_max’ variable indicates the fact that the higher the maximum speed, the shorter the duration is at each risk level of driving behavior. This is an expected result, because drivers that were included in the experiment are experienced drivers and thus can handle higher speeds adequately to reduce their safety level [55]. On the contrary, the positive coefficient of the ‘Distance travelled_sum’ variable indicates that the longer the distance travelled, the longer the duration is at each safety level. However, as described in previous sections regarding the attributes of the models, the minimum value of Distance travelled_sum indicates a non-significant contribution of this variable to the prediction process. Nevertheless, the fact that with longer distance travelled, the safety level remains intact denotes the impact of driver fatigue on driving risk. 

As stated in the literature review, to our knowledge, a similar development of the above approach has not been found in research. However, a similar methodology is applied to short-term traffic prediction problems. Comparing the results of this study with other findings in the literature, it appears that in general Elastic Net model [27] and Lasso model [26] both have high performance as the coefficient of determination R^2^ in this research, and those in the literature are relatively similar. However, in order to be able to examine the in-depth performance of the regression models, it is necessary to consider additional measures such as the mean absolute error (MAE) and the mean absolute percentage error (MAPE) [56] in the future.

## 6. Conclusions

This paper aimed to propose a framework for identifying the risk level of driving behavior and predicting the duration of driving at each safety level. An important step was the definition of driving behavior risk levels. Among the techniques examined, the definition of levels based on specific thresholds of time headway provides results relevant to the literature regarding the distribution of samples in the classes. To avoid bias in the models, the variables of time headway and time to collision were not taken into account during the classification process, excluding two very important risk factors. In the future, it is necessary to examine alternative methods of determining the risk levels of driving behavior to examine more risk factors.

Through the identification of risky driving behavior level processes, four classification algorithms were developed of which the Random Forest and Multilayer Perceptron outperformed the Support Vector Machines and AdaBoost classifiers. The two models (RF & MLP) were found to have a high capability of identifying all risk levels of driving behavior.

In the effort of improving the performance of the models, a feature selection was performed utilizing the feature importance as well as their correlation. Through the process of calculating the feature importance, it emerged that distance travelled, speed, and the speed limit are significant in identifying the risk level of driving behavior. In contrast, the variables FatigueEvent and HandsOnEvent were not particularly important during the classification process. However, the driver’s condition and interaction with the steering wheel are directly related to other driving factors such as speed or distance traveled.

In addition to the development of classification models, this research also deals with the unequal distribution of samples in the classes using the ADASYN resampling method. The main advantage of ADASYN is that the algorithm doesn’t copy the same minority data; instead, more data is generated for examples that are harder to learn. This is the first time that ADASYN is combined with a variety of machine learning classifiers for the real-time safety assessment of highly disaggregated driving behavior data. 

In the second part of the study, three regression algorithms were developed to predict the duration that each driver spends at each safety level. Through regression process, it was found that among all the examined variables, the maximum speed and total distance travelled provided statistically significant results. Based on the coefficients, maximum speed has the main, negative effect on driving duration at different safety levels. Ridge, Lasso, and Elastic Net Regression are using L1 and L2 regularization, reducing the size of coefficients for not useful variables, and performing some kind of ‘Feature Selection’. Therefore, maximum speed is particularly important in predicting driving duration at each level. It should also be mentioned that to the best of the knowledge of the authors, a combined approach for detecting not only the safety level of a driver but also the duration of each level, has not been published yet. This fact forms another novelty of the current study. 

Nevertheless, future studies could examine deep learning models (such as Convolutional Neural Networks [56,57] and Long Short-Term Memory (LSTM) [56,58]) which, based on relevant research, tend to perform better. Furthermore, a larger dataset and naturalistic driving data would also enhance the study results. However, due to processing power and time limitations, these analyses could not be performed at the time of this research. 

## Figures and Tables

**Table 1:** Different scenarios applied during the driving simulator experiment.

Scenario	Road Section	Number of Lanes	Speed Limits
A	0–6300 m	1 × 1	70 km/h
	6300–11,300 m	2 × 2	90 km/h
	11,300–16,500 m	2 × 2	120 km/h
B	0–6100 m	2 × 2	90 km/h
	6100–12,000 m	2 × 2	120 km/h
	12,000–18,200 m	1 × 1	70 km/h
C	0–6000 m	2 × 2	120 km/h
	6000–11,000 m	2 × 2	90 km/h
	11,000–17,200 m	1 × 1	70 km/h

**Table 2:** Description of variables collected by the driving simulator experiment.

Variable	Description	Units	Type
TTC	Time to collision with the vehicle ahead	Seconds	Numeric
Headway	Time headway to the vehicle ahead in the same lane	Seconds	Numeric
Speed	Vehicle speed	Kilometers per hour	Numeric
Distance_travelled	Distance driving	Meters	Numeric
BSAV_SpeedLimitKPH	Current speed limit	Kilometers per hour	Numeric
HandsOnEvent	Whether hands are on the steering wheel	None/both	Discrete
FatigueEvent	KSS score	32–35–39	Discrete

**Table 3:** Comparison of results of different methods for determining safety levels.

Technique	Risk Level of Driving Behavior		
	Normal	Dangerous	Avoidable Accident
K-means Clustering	239	1483	1599
Hierarchical Clustering	368	1204	1749
Threshold of the variable TTC_mean	3150	35	136
Threshold of the variable Speed_mean	3320	1	0
Threshold of the variable Headway_min	2820	338	163

**Table 4:** Descriptive statistics for input variables.

Variable	Description	Mean	St. Dev.	Min	Max
Speed_max	Maximum value of Speed variable for an interval of 30 s (km/h)	75.45	3.00	64.00	100.00
Distance travelled_sum	Sum of Distance travelled variable for an interval of 30 s (m.)	7,006,041.28	4,176,949.80	363.50	20,023,055.37
BSAV_SpeedLimitKPH_max	Maximum value of BSAV_SpeedLimitKPH variable for an interval of 30 sec (km/h)	95.94	20.96	75.50	125.50

**Table 5:** Classification metrics for the developed classifiers.

Classifier	Accuracy	Precision	Recall	False Alarm Rate	f1-Score
SVM	68.67%	51.35%	74.72%	12.47%	53.22%
RF	84.00%	59.41%	70.27%	11.47%	63.42%
AdaBoost	75.08%	52.31%	70.71%	11.30%	55.87%
MLP	81.28%	57.51%	72.04%	11.37%	61.79%

**Table 6:** Summary of Ridge Regression model.

Summary of Ridge Regression Model				
Coefficients:				
	Estimate	Std. Error	t Value	*p*-Value
Intercept	9966.72	472.91	21.08	0.00
Speed_max	−112.01	2.18	−51.44	0.00
Distance travelled_sum	0.01	0.00	8.90	0.00
R^2^ = 0.85		Adjusted R^2^ = 0.85		

**Table 7:** Summary of Lasso Regression model.

Summary of Lasso Regression Model				
Coefficients:				
	Estimate	Std. Error	t Value	*p*-Value
Intercept	9966.36	472.91	21.08	0.00
Speed_max	−112.02	2.18	−51.45	0.00
Distance travelled_sum	0.01	0.00	8.90	0.00
R^2^ = 0.85		Adjusted R^2^ = 0.85		

**Table 8:** Summary of Elastic Net Regression model.

Summary of Elastic Net Regression Model				
Coefficients:				
	Estimate	Std. Error	t Value	*p*-Value
Intercept	9697.04	472.98	20.46	0.00
Speed_max	−108.84	2.18	−49.87	0.00
Distance travelled_sum	0.01	0.00	8.96	0.00
R^2^ = 0.85		Adjusted R^2^ = 0.85		

## Data Availability

Not applicable.
